# Improving Meal Acceptance of Individuals With Autism Spectrum Disorder (AUT-MENU Project): Protocol for a Bicentric Intervention Study

**DOI:** 10.2196/57507

**Published:** 2025-05-21

**Authors:** Maria Vittoria Conti, Chiara Breda, Sara Basilico, Ilaria Zambon, Angeliki Sofroniou, Stefania Ruggeri, Maria Luisa Scalvedi, Hellas Cena

**Affiliations:** 1 Laboratory of Dietetics and Clinical Nutrition, Department of Public Health, Experimental and Forensic Medicine University of Pavia Pavia Italy; 2 Research Centre for Food and Nutrition, CREA Rome Italy; 3 Clinical Nutrition Unit Istituti Clinici Scientifici Maugeri Istituto di Ricovero e Cura a Carattere Scientifico Pavia Italy; 4 Italian Institute for Planetary Health Milan Italy

**Keywords:** autism spectrum disorder, collective catering, parent training, nutrition, food selectivity

## Abstract

**Background:**

Individuals with autism spectrum disorder (ASD) often exhibit low dietary diversity due to food selectivity, leading to various forms of malnutrition, such as obesity or micronutrient deficiencies.

**Objective:**

The main objective of the AUT-MENU project is to improve meal acceptance among individuals with ASD. A secondary goal is to evaluate the effectiveness of a nutrition education course for parents of enrolled participants to reduce food selectivity.

**Methods:**

The study is a bicentric intervention conducted in 3 care centers (Pavia and Milan) and 1 secondary school (Rome), involving approximately 200 participants with ASD aged 3 to 35 years. The study consists of an observational phase (T0) and an intervention phase (T1). At T0, biographical data, clinical characteristics, and dietary patterns of participants are collected. Based on T0 findings and existing nutritional recommendations for individuals with ASD, targeted menus are developed and tested. At T1, the same assessment tools used at T0 will be applied to evaluate intervention effects. Additionally, a nutrition education course for caregivers will be implemented between T0 and T1, with a pre- and postcourse knowledge questionnaire to assess its effectiveness.

**Results:**

Due to different timelines depending on the centers and schools involved, participant enrollment and data collection will take place at different times between Pavia, Milan, and Rome. In September 2024, enrollment was held in the Pavia and Milan care centers for a total of 74 participants enrolled. In Rome, the enrollment phase has not yet started; activities are expected to be carried out similar to those in Pavia and Milan.

**Conclusions:**

The AUT-MENU study is expected to yield significant insights and improvements in meal acceptance among individuals with ASD, particularly through the introduction of targeted menus in collective catering settings both in care centers and schools.

**Trial Registration:**

ClinicalTrials.gov NCT06266377; https://clinicaltrials.gov/study/NCT06266377

**International Registered Report Identifier (IRRID):**

DERR1-10.2196/57507

## Introduction

### Background

In recent years, the scientific community has placed particular emphasis on investigating and elucidating the feeding-related behaviors of individuals diagnosed with autism spectrum disorder (ASD). Research has highlighted the rituals related to mealtime that are frequently observed among this population [1].

Individuals with ASD commonly present various feeding challenges, including food refusal, limited food intake, and behavioral difficulties during mealtime [2,3], which results in imbalanced food habits [4]. These challenges fall under the umbrella term of food selectivity (FS), a term also referred to in the literature as “picky eating” and “fussy eating” [4]. Specifically, FS is characterized by the rejection of specific types of food, whether familiar or unfamiliar, often refusing more foods than they accept [5]. This selectivity may involve entire food groups, such as fruits and vegetables, or foods that share specific sensory characteristics, including taste, texture, or odor. When FS persists over time, the individual can be classified as “picky eater,” which may impact dietary variety and nutritional intake [5].

Valenzuela-Zamora et al [3] reported a prevalence of 23% to 69% for FS among a sample of 247 children with ASD aged 2-18 years compared with 267 children with neurotypical condition, where the prevalence ranges from 1% to 37%.

Specifically, individuals with ASD and FS tend to gravitate toward foods with soft or semiliquid textures, pale hues, mild flavors, homogeneity of hues within the same dish, and dishes containing ingredients of similar, noncontrasting color tones, while they show reluctance toward foods that are bitter, sour, or spicy. Additionally, they often avoid strong colors, strong odors, and high temperatures, leading to the exclusion of entire food groups [1,6] (eg, canned fish or vegetables such as broccoli and cauliflower). Moreover, individuals with ASD exhibit a notable preference for processed foods high in energy, simple sugars, saturated fats, and salt but deficient in essential minerals and vitamins [1]. Such preferences may contribute to an increased risk of becoming overweight or developing obesity and micronutrient deficiencies [2].

In certain instances, it is possible to pinpoint physiological factors that directly or indirectly contribute to these distinct feeding challenges, such as compromised sensory processing, issues in oral motor skills (chewing and swallowing), and gastrointestinal disorders [4]. Conversely, when identifiable organic factors are absent, FS may be interpreted as an expression of the restricted interests and behavioral inflexibility frequently linked with ASD [4].

The evolution of feeding challenges with age remains a subject of contention in the literature. In the systematic review by Page et al [7], 7 studies are included, exploring the relationship between age and feeding difficulties. Among these studies, only 3 indicate amelioration in eating challenges over time, regardless of interventions specifically addressing FS [7]. Specifically, these 3 studies followed a cross-sectional model. Beighley et al [8] found that FS, defined as “will eat only certain food,” decreased with increasing age. Gray et al [9] compared eating behaviors among children with ASD from childhood to adolescence and found that parents reported that picky eating decreased and eating a variety of foods increased from early childhood to adolescence. Finally, Bandini et al [10] found that food refusal declined with age, but there was no significant change in food repertoire over the 6-year period.

Considering what has been described so far, the delicate nature of mealtime for individuals with ASD is clear, and it highlights the challenges of managing FS attitudes both in domestic and collective catering settings. Acting in the household environment may present a crucial window of opportunity since family mealtime is described by most parents as stressful and frustrating. Moreover, parents or caregivers may unintentionally exacerbate meal-related anxiety and stress through nonfunctional attitudes [2,11-13]. Therefore, it is essential to provide caregivers with valuable information to address the challenges that arise during these moments. The organization “Autism Speaks” has released the document “Parent’s Guide to Feeding Behavior in Children With Autism” [14], suggesting simple strategies that can be easily implemented at the domestic level.

Collective catering is a critical setting too, as many adults with ASD consume their meals in the care centers where they reside, while children and adolescents have at least 1 meal a day at school. This setting opens a potential opportunity for intervention that, if properly exploited, can enable an improvement in the nutritional status of individuals with ASD through specifically formulated menus, leading to a cascade of beneficial effects on their health [1]. There are currently no specific guidelines about how to customize collective catering menus for participants with ASD in Italy. In fact, due to the specificity of the target population and the complexity of working with individuals affected by ASD, no multicenter studies aimed at improving food acceptance have so far been carried out on large groups across different age ranges.

The authors designed and conducted a pilot study, the FOOD-AUT project [6], in which canteen menus were adapted to meet the nutritional and sensory needs of adults with ASD, with the aim of reducing their FS and consequently improving their health. The study involved 22 participants and focused on adapting meals based on key sensory characteristics, such as texture, color, and shape, to enhance food acceptance. The results showed that personalized meal modifications could positively influence dietary variety and meal consumption in individuals with ASD. Since the pilot study was conducted in a single setting with a limited sample size, this study, the AUT-MENU project (improving the meal acceptance of participants with ASD), aims to extend the same model to multiple contexts, including care facilities and schools, to further evaluate its effectiveness. This broader implementation will strengthen the dataset and provide more robust evidence, ultimately supporting the development of specific dietary guidelines for collective catering services tailored to individuals with ASD.

### Study Objectives

#### Primary Objectives

The primary objective of the AUT-MENU project is to assess meal consumption among individuals with ASD in collective catering settings both quantitatively (eg, portion intake) and qualitatively (eg, sensory characteristics influencing food acceptance, such as texture, color, smell, and shape). Based on these findings and existing dietary guidelines for individuals with ASD [1,6], the project aims to improve meal acceptance by adapting the proposed meals accordingly.

#### Secondary Objectives

The secondary objective is to evaluate the feasibility of a structured nutrition and behavioral education program for parents and caregivers of enrolled participants. This intervention aims to improve parents’ knowledge on FS and meal experience at home, complementing the modifications implemented in the collective catering settings.

## Methods

### Study Overview

The AUT-MENU study is a single-arm pre- and postbicentric intervention study (without control). The study is conducted by a multidisciplinary team consisting of researchers from the Laboratory of Dietetics and Clinical Nutrition of the University of Pavia and the Research Centre for Food and Nutrition of Rome.

### Setting

#### Overview

The study is being conducted in 2 different regions of Italy. In the northern region, 3 care centers have been involved: Tiglio Fondazione Onlus in Pavia, Dosso Verde in Milan, and Dosso Verde in Pavia. In the central region, the study is taking place in 1 high school in Rome: the “Giuseppe Garibaldi” Agricultural Technical Institute.

#### Tiglio Fondazione Onlus

Fondazione Tiglio is a nonprofit organization active since 2004. The center offers a range of residential and nonresidential services specifically designed for young adults or adults (≥18 years) with severe or profound intellectual, physical, and sensory disabilities as well as neurodevelopmental disorders (eg, autism). These services include educational, assistive, socio-health care support, work training, and recreational-cultural activities. The facility also provides an external catering service that offers a complete lunch for the users.

#### Dosso Verde Institute

The Dosso Verde Institute is a Neuropsychiatric Rehabilitation and Psychotherapy Center for Developmental Age. Founded in 1962, the institute provides residential, day, and outpatient services for minors (<18 years) with neurodevelopmental disorders, particularly ASD and intellectual disabilities as well as emotional and behavioral disorders, childhood and adolescent mental health issues, and learning disabilities. The Dosso Verde Institute has facilities in both Pavia and Milan. In the Pavia center, an external catering service is provided, while in Milan, meals are supplied by the internal kitchen of the primary school housed within the same building, “Regina Mundi.”

#### “Giuseppe Garibaldi” Agricultural Technical Institute

The “Giuseppe Garibaldi” Agricultural Technical Institute is a secondary school in Rome, established in 1872. The institute is dedicated to training agricultural experts and technicians knowledgeable in the natural environment. The institute is also home to a boarding school, which accommodates students, including those from nearby schools, who come from distant areas and offers a semiboarding option with lunch services and afternoon study sessions. In the school, meals are prepared daily by a collective catering company.

### Ethical Considerations

This study was approved by the Ethics Committee of the Department of the Nervous System and Behavioral Sciences in January 2024 (approval 155/23). The study adheres to all institutional guidelines and ethical principles outlined in the Declaration of Helsinki. Parents or legal guardians of all participants provided written informed consent before enrollment. They will receive detailed explanations regarding the study objectives, procedures, and potential risks and benefits. Participants will have the right to withdraw from the study at any time without consequences. All collected data will be anonymized and deidentified to protect participant privacy. Identifiable information will be securely stored and accessed only by authorized research personnel. Data will be reported in an aggregate format, ensuring that individual participants cannot be identified.

### Selection Criteria

The target population for the study adheres to the inclusion and exclusion criteria listed in Textbox 1.

**Inclusion and exclusion criteria.**

**Inclusion criteria**

- Presence in medical records of a diagnosis of autism spectrum disorder (ASD; according to
  DSM-5 [Diagnostic and Statistical Manual of Mental Disorders {Fifth Edition}] diagnostic criteria) including all the 3 severity levels [15].
- Being a patient of Tiglio Fondazione Onlus in Pavia, Dosso Verde in Milan, and Dosso Verde in Pavia or a student of “Giuseppe Garibaldi” Agricultural Technical Institute in Rome.
- Children and adolescents aged 3 to 17 years from the Dosso Verde centers in Milan and Pavia, young adults aged 18 to 35 years from Tiglio Fondazione Onlus (Pavia), and high school students aged 14 to 19 years from the secondary school “Giuseppe Garibaldi” Agricultural Technical Institute.
- Signed informed consent from parents or legal guardians is required for each respondent.

**Exclusion criteria**

- Participants who are unable to feed themselves independently due to physical or cognitive impairments.
- Participants with allergies, intolerances, or conditions requiring a specialized diet.
- Participants with other neurological or medical conditions (eg, diabetes and epilepsy) that may affect food consumption or require specific medical attention, excluding ASD.

### Recruitment

In Pavia and Milan, care centers with collective catering services were selected, while in Rome, schools offering afternoon activities—including lunch—were chosen. Researchers directly contacted care centers for individuals with ASD and schools offering lunch to present the project. Interested centers in Pavia and a high school in Rome participated in multilevel project presentation sessions. Initially, events targeted health care professionals (child neuropsychiatrists, therapists, and educators) and school staff, followed by meetings with parents. These sessions informed and trained professionals who would act as project supporters and addressed parents’ questions. During parent meetings, informed and privacy consent were collected from parents or legal guardians.

### Study Design

#### Overview

**Figure 1:**
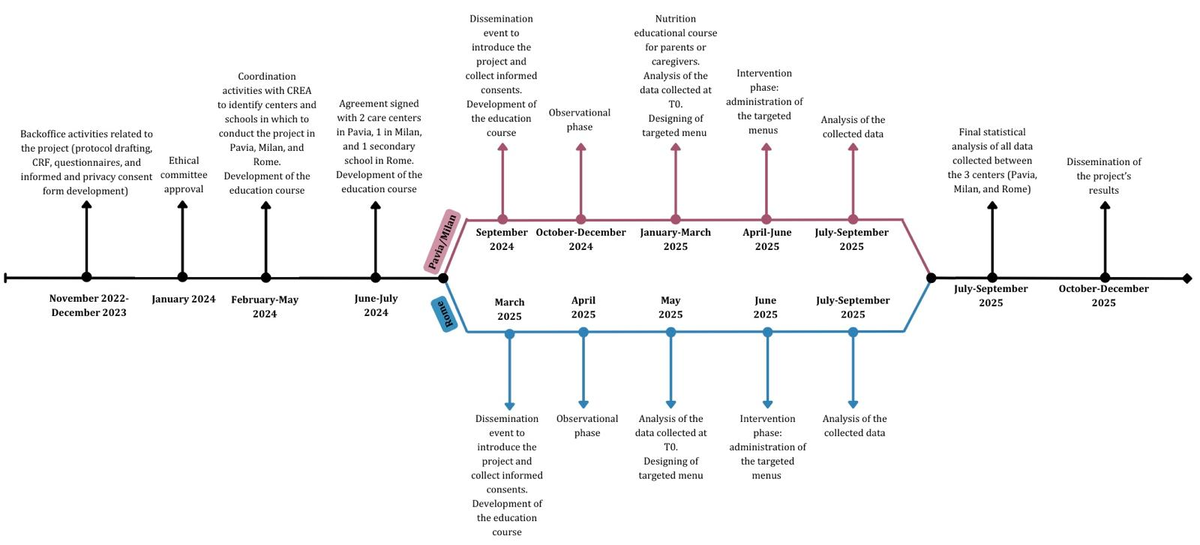
Study timeline. CREA: Research Centre for Food and Nutrition; CRF: case report form.

The study will last 36 months (November 2022-2025), including a 12-month experimental phase, from September 2024 until September 2025. Figure 1 shows the study timeline.

#### Meal Assessment and Menu Adaptation

The study was structured into 3 main phases: scouting, observation, and intervention. After enrolling the participating centers, researchers conducted a preliminary scouting phase by visiting care centers and schools to gain an in-depth understanding of lunchtime dynamics. This phase involved analyzing food service methods, types of cutlery used, staff interaction with participants, and portion sizes served. Based on these observations, portion sizes were standardized according to age groups to ensure consistency in meal assessment and prevent potential biases in data collection. The observation phase (T0) aimed to assess food consumption from standard menus during lunchtime, focusing on both quantity and quality aspects such as sensory characteristics such as shape, color, smell or taste, texture, and temperature. To systematically evaluate these aspects, the pictorial dietary assessment tool (PDAT) was used [16]. Each enrolled participant’s meal was assessed over 3 nonconsecutive days within a 4-week period, with additional sessions scheduled for those absent to ensure complete data collection. A total of 12 PDATs were collected for each participant. To minimize observer influence and reduce bias, researchers passively observed the participants during lunch without direct interaction. They were responsible for completing the PDATs based on observed consumption patterns. To enhance the accuracy of qualitative data, researchers also tasted and photographed each dish to capture its sensory characteristics comprehensively. At the end of each survey day, the research team convened to align on the organoleptic properties of the meals, ensuring consistency in data interpretation. Data entry was performed daily by 3 researchers (CB, SB, and IZ), who decoded the PDATs using a predefined classification system and entered the information into a Microsoft Excel spreadsheet for further analysis. The collected data will serve to compare consumption patterns with sensory characteristics in order to identify key factors influencing meal acceptance. This information will then be used to collaborate with mass catering companies to create dishes aligned with the preferences of individuals with ASD. The authors expect that the sensory modifications will align with those implemented during the FOOD AUT pilot study [6], specifically short pasta shapes (eg, bucatini, penne, orecchiette, and farfalle); foods with low-intensity colors, such as white and beige; uniform hues within the same dish; dishes composed of ingredients with similar, noncontrasting color tones; vegetables that are more readily accepted than orange ones; soft or semisoft textures, avoiding foods that are difficult to chew; mild, nonpungent odors, avoiding strong-smelling foods like certain fish species (eg, mackerel); and delicate flavors, avoiding bitterness and sourness, with reduced use of strong spices, garlic, and onion.

However, the authors consider that having a larger sample than in the pilot study, the most accepted sensory characteristics might be slightly different. The intervention phase (T1) will involve introducing these modified menus and repeating the same assessment process used during T0. Each enrolled participant will undergo the same PDAT-based evaluation over 12 meals to determine whether the targeted menus are better received and lead to increased food consumption.

#### Nutrition Education Course for Parents and Caregivers

Between the T0 and T1 phases, a digital nutrition education course will be offered to subgroups of parents and caregivers of enrolled participants in the Milan and Pavia centers. The course aims to reduce meal-related behavioral difficulties, including FS in individuals with ASD. Participation will be voluntary, following an informational session on the project’s objectives. Designed based on healthy eating guidelines and scientific literature on ASD and nutrition, the course is led by a multidisciplinary team, including a dietitian, nutrition biologists, speech therapists, and a psychologist. It consists of 7 modules delivered through weekly 1-hour sessions, integrating theoretical explanations with practical demonstrations using videos, images, and digital activities. The topics covered in the modules are as follows:

- Module 1: the role of nutrition on health and macro- and micronutrients.
- Module 2: FS: associated factors and consequences.
- Module 3: healthy plate tailored to the needs of individuals with ASD.
- Module 4: behavioral strategies for the consumption of nonpreferred foods.
- Module 5: sensory strategies for the consumption of nonpreferred foods.
- Module 6: environment and meal presentation.
- Module 7: mother-child relationship.

To assess the course’s impact and feasibility, specific evaluation tools (detailed in the following section) will be used, along with the recording of attrition rates throughout the program.

### Data Collection

#### Overview

Details of data collected from both participants and their families throughout the study period are presented in the following subsections and summarized in Table 1. Data regarding sociodemographic information, food habits, and feeding behaviors during mealtime were collected through 20- or 25-minute telephone interviews, conducted by a researcher. Data entry is carried out in parallel with data collection, through the daily entry of anonymized data into a preset database.

**Table 1:** Summary of data collection tools.

Tool	Evaluated aspects	Addressed to	Filled out by	T0	T1
CRF^a^	General information of enrolled participants and their families	Parents or caregivers	Researcher (telephone interview)	✓	
KIDMED^b^ [17] (3-24 years) and MEDQS^c^ [18] (25-35 years)	Food habits and adherence to the Mediterranean diet	Parents or caregivers	Researcher (telephone interview)	✓	
BAMBI^d^ [19] (3-11 years) and STEP^e^ [20] (12-35 years)	Risk of food selectivity	Parents or caregivers	Researcher (telephone interview)	✓	
PDTA^f^ [16]	Quantitative and qualitative food consumption	Enrolled participants	Researchers (in presence)	✓	✓
Nutrition knowledge questionnaire	Nutrition course knowledge	Parents or caregivers	Researcher (telephone interview)	Before and after the course	Before and after the course
Satisfaction questionnaire	Nutrition course satisfaction	Parents or caregivers	Researcher (telephone interview)	After the course	After the course

^a^CRF: case report form.

^b^KIDMED: Mediterranean Diet Quality Index for Children and Adolescents.

^c^MEDQS: New Validated Short Questionnaire for the Evaluation of the Adherence of Mediterranean Diet and Nutrition Sustainability in all adult population groups.

^d^BAMBI: Brief Autism Mealtime Behavior Inventory.

^e^STEP: Screening Tool of Feeding Problems.

^f^PDAT: pictorial dietary assessment tool.

#### Sociodemographic Information

Sociodemographic and socioeconomic information of the participants were collected at T0 in a case report form (Multimedia Appendix 1), which was divided into 2 parts. The first part collects data about the participants with ASD: gender, age, ethnicity, and clinical information (drug therapy, therapeutic paths, anthropometric measurements, and blood values), while the second part of the questionnaire aims to gather information about the primary parent, collecting marital status, age, ethnicity, educational level, and therapeutic approaches for managing FS.

#### Food Habits

Information regarding the food habits was assessed at T0. Specifically, the Mediterranean Diet Quality Index for Children and Adolescents questionnaire [17] and the New Validated Short Questionnaire for the Evaluation of the Adherence of Mediterranean Diet and Nutrition Sustainability in all adult population groups questionnaire [18] (Multimedia Appendices 2 and 3, respectively) were used to assess adherence to the Mediterranean diet and to get an overview of the consumption of the main food groups (fruits, vegetables, pasta and cereals, legumes, fish, meat, and dairy products).

#### Feeding Behaviors During Mealtime

Information regarding feeding behaviors during mealtime was assessed at T0. Specifically, Brief Autism Mealtime Behavior Inventory questionnaire [19,20], validated for a population of 3-11 years, and Screening Tool of Feeding Problems questionnaire [21], validated for a population of 12-99 years (Multimedia Appendices 4 and 5, respectively) were used to evaluate dysfunctional meal-related behaviors, including FS of participants with ASD.

#### Meal Assessment

The quantity and quality assessment of meals consumed during lunchtime are conducted using the PDAT [16] at both T0 and T1. Rather than relying on the gold standard weighing method, the PDAT offers a less invasive alternative, allowing for uninterrupted mealtime evaluation. Additionally, when compared to the gold standard, it has demonstrated high accuracy and strong correlations with macronutrient and micronutrient intake [16]. The PDAT consists of 2 sections (Multimedia Appendix 6): a quantitative and a qualitative component, with the latter developed by researchers based on findings from the FOOD-AUT project [6]. The quantitative section enables the monitoring of each meal component (first course, second course, side dish, accompaniment—bread, breadsticks, and crackers—dessert, fruit, yogurt, and beverages) by recording the amount consumed. To facilitate data collection, intake is recorded both numerically (empty plate, one-fourth, half, three-fourth, and fully consumed) and graphically. The graphic representation uses 4 circles (representing a plate divided into 4 sections), which range from white (not consumed) to blue (completely consumed). For beverages, a stylized glass is used, with a gradually increasing water fill indicating empty, one-quarter full, half full, three-quarters full, and full consumption. The qualitative section evaluates the sensory characteristics of each meal component, detailing its shape, color, smell or taste, texture, and temperature. The development of the qualitative part of the PDAT was finalized with the collaboration of a sensory expert, who helped define the possible alternatives for completing each of the main organoleptic characteristics.

#### Nutrition Knowledge

Nutrition knowledge will be assessed in parents and caregivers participating in the nutrition education course. A questionnaire, designed based on the course topics, consists of 21 questions (3 per module; Multimedia Appendix 7). Researchers will administer the questionnaire via telephone interviews before and after the course to evaluate knowledge acquisition.

#### Satisfaction

Satisfaction will be evaluated in parents and caregivers attending the course through a 13-question questionnaire (Multimedia Appendix 8). Administered at the end of the program via telephone interviews, it aims to assess the course’s usefulness, organization, and areas for improvement. Table 1 below summarizes which tools are administered, to whom, with what objective, and at what stage of the project.

### Outcomes and Statistical Analysis

The main outcome of the study testing the improvement of the meal acceptance is an increased food consumption of the proposed targeted meals. Specifically, food acceptance of targeted menus will be calculated as the average intake of the 12 administered set meals, ranging from 0 to 1, comparing results collected at T0 and at T1. The improved food acceptance associated with targeted menus will be estimated assuming a threshold of acceptance of 0.6, calculated as the percentage of cases showing an average PDAT lower than that value. Since the results of the pilot study were encouraging [6], showing an average PDAT greater than or equal to 0.8 in 50% (n=22) of participants, a threshold of 0.6 is assumed as a precautionary measure due to the variability in standard meal composition.

Multiple correspondence analysis at T0 was used to study the association between specific sensory aspects and food consumption, categorized into 5 levels (1=no consumption to 5=total consumption). The analysis produced a map showing the distances between the categories of the qualitative variables and food consumption levels.

Concerning the subgroup of caregivers attending the nutrition education course, statistical analysis will focus on effectiveness evaluation of the course, measured by the comparison of nutrition knowledge score, the prevalence of FS score, and the adherence to the Mediterranean diet score before and after the education intervention and by the dropout rate at the end of the course.

Since no preliminary information is available on the average consumption and variability of the PDAT, the determination of sample size is made on the expectation of a possible decreased propensity of FS. FS is an attitude found in 80% of the population with ASD, starting as early as childhood [6]. Hence, setting a significant level of α=.05 and a test power of 90%, under the assumption that the rate of FS may decrease to 15 percentage points, namely from p(T0)=80% to p(T1)=65%, 150 participants could be sufficient [22].

## Results

Due to different timelines depending on the centers and schools involved, participant enrollment and data collection will take place at different times between Pavia, Milan, and Rome. In September 2024, enrollment was held in the Pavia and Milan care centers for a total of 74 participants enrolled. The observation phase took place between October and December 2024 for a total of 4 weeks for each center, while the intervention phase is scheduled between April and June 2025. In Rome, the school in which to carry out the study has been selected, but the enrollment phase has not yet started; activities are expected to be carried out similar to those in Pavia and Milan.

## Discussion

### Principal Findings

The AUT-MENU study is expected to yield significant insights and improvements in meal acceptance among individuals with ASD, particularly through the introduction of targeted menus in collective catering settings. Specifically, the study anticipates that individuals with ASD will demonstrate an increased acceptance of and preference for the targeted menus compared to the standard ones, as evidenced by the FOOD-AUT pilot study [6]. By using PDAT to assess food consumption quantitatively and qualitatively, the study aims to identify and leverage the sensory characteristics of food that are most conducive to acceptance by individuals with ASD. These findings will provide valuable evidence for tailoring collective catering practices to better accommodate this population’s specific dietary preferences and needs.

A notable outcome of the study is the expected reduction in FS and meal-related behavioral challenges, particularly in the subgroup of participants whose caregivers participate in the nutrition education course. The course is designed to equip caregivers with strategies to address FS effectively, promote healthier eating patterns, and improve the overall mealtime environment. It is anticipated that caregivers will demonstrate increased nutrition knowledge and confidence in implementing these strategies, leading to observable improvements in the dietary habits of their children or dependents with ASD.

On a broader scale, the study is expected to highlight the feasibility of implementing customized dietary interventions in collective catering environments. This includes the ability of catering services to target menus based on sensory preferences and the willingness of staff at care centers and schools to implement these changes. By working collaboratively with catering operators and using sensory-focused strategies, the study anticipates not only improved food consumption but also a better overall dining experience for individuals with ASD.

Improvements are expected also in caregivers’ nutrition knowledge scores, FS prevalence scores, and adherence to the Mediterranean diet thanks to the educational course. These outcomes will highlight the importance of a multidisciplinary approach, involving dietitians, psychologists, speech therapists, and child neuropsychiatrists in addressing the complex dietary challenges faced by this population.

Finally, the AUT-MENU study is poised to contribute to the limited body of research on ASD and dietary interventions in collective catering settings. By disseminating findings through academic publications, conferences, and community outreach, the study aims to provide a replicable framework for similar interventions in other regions and contexts. While the study acknowledges limitations, such as the lack of representativeness of the sample and the absence of national prevalence data on ASD, the anticipated results will provide a critical foundation for future research and policy development, ultimately improving the nutritional health and quality of life for individuals with ASD.

### Strengths and Limitations

The AUT-MENU study is unique in that it intervenes at the level of collective catering to improve the mealtime experience for individuals with ASD, an area that has not been explored previously. The intervention takes a holistic approach, addressing both collective catering and the domestic context to enhance participants’ diets. To ensure that the mealtime experience of the enrolled participants remains undisturbed, the study uses validated and accurate noninvasive tools. However, a limitation of the study is the lack of a comprehensive national and subnational prevalence study on individuals with ASD in Italy, which affects the representativeness of the sample. The sample population is not representative of the ASD population in Italy, as the sample size could not be estimated based on the actual prevalence of ASD.

## Appendix

Multimedia Appendix 1 Case report form.

Multimedia Appendix 2 Mediterranean Diet Quality Index in Children and Adolescents.

Multimedia Appendix 3 New Validated Short Questionnaire for the Evaluation of the Adherence of Mediterranean Diet and Nutrition Sustainability in all adult population groups.

Multimedia Appendix 4 Brief Autism Mealtime Behavior Inventory.

Multimedia Appendix 5 The Screening Tool of Feeding Problems.

Multimedia Appendix 6 Pictorial dietary assessment tool.

Multimedia Appendix 7 Nutrition knowledge questionnaire.

Multimedia Appendix 8 Satisfaction questionnaire.

## Abbreviations

ASD: autism spectrum disorder
FS: food selectivity
PDAT: pictorial dietary assessment tool

## Data Availability

The datasets generated or analyzed during this study are not publicly available due to privacy reasons but are available from the corresponding author on reasonable request.
