# Supplementary material for: Improving Meal Acceptance of Individuals With Autism Spectrum Disorder (AUT-MENU Project): Protocol for a Bicentric Intervention Study
Source: JMIR Res Protoc. 2025 May 21;14:e57507. doi: 10.2196/57507 (PMC12138289; doi:10.2196/57507)
Supplement: Multimedia Appendix 1 [file resprot_v14i1e57507_app1.docx]

| INFORMATION ABOUT YOUR SON/DAUGHTER | |
| --- | --- |
| 1) Name and surname: |  |
| 2) Date of birth: |  |
| 3) Sex | □ M  □ F |
| 4) Ethnicity | □ African  □ Afro-american  □ Asian  □ Caucasic  □ Hispanic |
| 5) Nationality: |  |
| 6) Has your son/daughter attended and completed therapy for food selectivity with a professional (speech therapist / psychomotricist / psychologist / ABA technician) in the past? | □ Yes  □ No |
| 7) If yes, how long has he/she been finishing it? | □ Less than 1 year  □ More than 1 year |
| 8) Is your son/daughter currently undergoing therapy for the management of food selectivity with a professional (speech therapist / psychomotricist / psychologist /ABA technician)? | □ Yes  □ No |
| 9) If yes, for how long? | □ Less than 1 year  □ More than 1 year |
| 10) Is your son/daughter currently taking medications and/or supplements? | □ Yes  □ No |
| 11) If yes, please indicate which one(s): __________________________________________________________________ | |
