# Supplementary material for: Improving Meal Acceptance of Individuals With Autism Spectrum Disorder (AUT-MENU Project): Protocol for a Bicentric Intervention Study
Source: JMIR Res Protoc. 2025 May 21;14:e57507. doi: 10.2196/57507 (PMC12138289; doi:10.2196/57507)
Supplement: Multimedia Appendix 2 [file resprot_v14i1e57507_app2.docx]

| 1) Does your son/daughter consume a fruit or juice every day? | Yes | No |
| --- | --- | --- |
| 2) Does your son/daughter consume a second fruit every day? | Yes | No |
| 3) Does your child consume vegetables, cooked or raw, regularly, once a day? | Yes | No |
| 4) Does your child consume vegetables, cooked or raw, more than once a day? | Yes | No |
| 5) Does your child consume fish regularly (at least 2-3 times a week)? | Yes | No |
| 6) Does your son/daughter go to fast food (hamburgers) more than once a week? | Yes | No |
| 7) Does your son/daughter appreciate legumes and consume them more than once a week? | Yes | No |
| 8) Does your son/daughter consume pasta and rice every day (5 or more times a week)? | Yes | No |
| 9) Does your son/daughter consume cereal grains or bread for breakfast? | Yes | No |
| 10) Does your son/daughter consume nuts regularly (at least 2-3 times a week)? | Yes | No |
| 11) Is olive oil used at home in the preparation of your son/daughter's consumed dishes? | Yes | No |
| 12) Does your son/daughter skip breakfast? | Yes | No |
| 13) Does your son/daughter consume dairy products for breakfast (yogurt, milk, etc.)? | Yes | No |
