# Supplementary material for: Improving Meal Acceptance of Individuals With Autism Spectrum Disorder (AUT-MENU Project): Protocol for a Bicentric Intervention Study
Source: JMIR Res Protoc. 2025 May 21;14:e57507. doi: 10.2196/57507 (PMC12138289; doi:10.2196/57507)
Supplement: Multimedia Appendix 3 [file resprot_v14i1e57507_app3.docx]

| How Many Portions of These Food Groups Did You Have Last Month? | | | |
| --- | --- | --- | --- |
| Cereals & cereal products (including whole, sweets excluded) 130 g | <1 portion/day | 1–1.5 portion/day | >1.5 portion/day |
| Legumes 70 g | <1 portion/week | 1–2 portion/week | >2 portion/week |
| Fresh vegetables 100 g | <1 portion/day | 1–2.5 portion/day | >2.5 portion/day |
| Fresh fruit 150 g | <1 portion/day | 1–2 portion/day | >2 portion/day |
| Dairy products 180 g | <1 portion/day | 1–1.5 portion/day | >1.5 portion/day |
| Fresh fruit 150 g | <1 portion/week | 1–2.5 portion/week | >2.5 portion/week |
| Fish & fish products (except shellfish and crustaceans) 100 g | <1 portion/week | 1–2.5 portion/week | >2.5 portion/week |
| Meat & meat products 80 g | <1 portion/day | 1–1.5 portion/day | >1.5 portion/day |
| Olive oil | Occasional Consumption (<5 spoons/day) | Regular Consumption (about 4–5 spoons/day) | Frequent Consumption (>4 spoons/day) |
