# Supplementary material for: Improving Meal Acceptance of Individuals With Autism Spectrum Disorder (AUT-MENU Project): Protocol for a Bicentric Intervention Study
Source: JMIR Res Protoc. 2025 May 21;14:e57507. doi: 10.2196/57507 (PMC12138289; doi:10.2196/57507)
Supplement: Multimedia Appendix 4 [file resprot_v14i1e57507_app4.docx]

|  | **Never or rarely** | **Sometimes** | **Occasionally** | **Often** | **Almost always** |  | **Do you think it's a problem?** | |
| --- | --- | --- | --- | --- | --- | --- | --- | --- |
| 1. My child cries or screams during mealtimes |  |  |  |  |  |  | Yes | No |
| 2. My child turns his/her face or body away from food |  |  |  |  |  |  | Yes | No |
| 3. My child remains seated at the table until meal is finished |  |  |  |  |  |  | Yes | No |
| 4. My child expels food that he/she has eaten |  |  |  |  |  |  | Yes | No |
| 5. My child is aggressive during mealtimes |  |  |  |  |  |  | Yes | No |
| 6. My child displays self-injurious behavior during mealtimes |  |  |  |  |  |  | Yes | No |
| 7. My child is disruptive during mealtimes |  |  |  |  |  |  | Yes | No |
| 8. My child Closes mouth tightly when food is presented |  |  |  |  |  |  | Yes | No |
| 9. My child is flexible about mealtime routines |  |  |  |  |  |  | Yes | No |
| 10. My child is willing to try new foods |  |  |  |  |  |  | Yes | No |
| 11. My child dislikes certain foods and won’t eat them |  |  |  |  |  |  | Yes | No |
| 12. My child refuses to eat foods that require a lot of chewing |  |  |  |  |  |  | Yes | No |
| 13. My child prefers the same foods at each meal |  |  |  |  |  |  | Yes | No |
| 14. My child prefers ‘‘crunchy’’ foods |  |  |  |  |  |  | Yes | No |
| 15. My child accepts or prefers a variety of foods |  |  |  |  |  |  | Yes | No |
| 16. My child prefers to have food served in a particular way |  |  |  |  |  |  | Yes | No |
| 17. My child prefers only sweet foods |  |  |  |  |  |  | Yes | No |
| 18. My child prefers food prepared in a particular way |  |  |  |  |  |  | Yes | No |
