# Supplementary material for: Improving Meal Acceptance of Individuals With Autism Spectrum Disorder (AUT-MENU Project): Protocol for a Bicentric Intervention Study
Source: JMIR Res Protoc. 2025 May 21;14:e57507. doi: 10.2196/57507 (PMC12138289; doi:10.2196/57507)
Supplement: Multimedia Appendix 5 [file resprot_v14i1e57507_app5.docx]

|  | **Not at all** | **One to ten times per month** | **More than ten times per month** |
| --- | --- | --- | --- |
| 1) My son/daughter cannot independently feed |  |  |  |
| 2) Problem behaviors increase during meals |  |  |  |
| 3) My son/daughter does not demonstrate ability to chew |  |  |  |
| 4) My son/daughter chokes on food |  |  |  |
| 5) My son/daughter does not demonstrate ability to swallow |  |  |  |
| 6) My son/daughter will only eat select types of foods |  |  |  |
| 7) My son/daughter steals or attempts to steal food |  |  |  |
| 8) My son/daughter requires special equipment to feed |  |  |  |
| 9) My son/daughter attempts to eat non-food items |  |  |  |
| 10) My son/daughter prefers certain settings for eating |  |  |  |
| 11) My son/daughter only eats a small amount of food presented |  |  |  |
| 12) My son/daughter will continue to eat as long as food presented |  |  |  |
| 13) My son/daughter spits out food before swallowing |  |  |  |
| 14) My son/daughter steals of attempts to steal food outside mealtimes |  |  |  |
| 15) My son/daughter eats large amounts in short time |  |  |  |
| 16) My son/daughter requires special positioning during feeding |  |  |  |
| 17) My son/daughter swallows without chewing sufficiently |  |  |  |
| 18) My son/daughter regurgitates or re-swallows food |  |  |  |
| 19) My son/daughter pushes food away or attempts to leave area |  |  |  |
| 20) My son/daughter only eats foods at certain temperature |  |  |  |
| 21) My son/daughter vomits during or right after meals |  |  |  |
| 22) My son/daughter prefers specific feeder |  |  |  |
| 23) My son/daughter only eats certain textures |  |  |  |
