# Supplementary material for: Improving Meal Acceptance of Individuals With Autism Spectrum Disorder (AUT-MENU Project): Protocol for a Bicentric Intervention Study
Source: JMIR Res Protoc. 2025 May 21;14:e57507. doi: 10.2196/57507 (PMC12138289; doi:10.2196/57507)
Supplement: Multimedia Appendix 7 [file resprot_v14i1e57507_app7.docx]

| SELECT THE CORRECT ANSWER FOR EACH QUESTION | | | |
| --- | --- | --- | --- |
|  | **A** | **B** | **C** |
| 1) Macro and micronutrients are: | **Macronutrients: carbohydrates, proteins, lipids; micronutrients: vitamins and minerals** | Macronutrients: carbohydrates and proteins; micronutrients: vitamins | Macronutrients: lipids and proteins; micronutrients: carbohydrates |
| 2) The main function of carbohydrates within our body is: | Enter into tissue composition | **Provide immediate energy** | To transport oxygen |
| 3) The main function of proteins within our body is: | Thermal insulation | Providing immediate energy | **Enter into the composition of tissues** |
| 4) Factors that may contribute to the development of food selectivity are: | **Sensory factors, behavioral factors, and organic factors (e.g., gastrointestinal disorders, gastroesophageal reflux)** | Organic factors | Behavioral factors |
| 5) Overweight and obesity are associated with: | Caloric deficiency | **Caloric excess** | Consumption of fiber-rich foods (vegetables, legumes) |
| 6) Diabetes and hypertriglyceridemia are generally associated with: | Consumption of fiber-rich foods | Consumption of protein-rich foods | **High consumption of foods high in simple sugars** |
| 7) The Harvard University healthy plate consists of: | **½ plate: vegetables and fruits; ¼ plate: healthy protein; ¼ plate: grains; dressing: sources of good fats** | ½ plate: grains; ¼ plate: healthy protein; ¼ plate: vegetables and fruits; topping: sources of saturated fat | ½ dish: healthy protein; ¼ dish: grains; ¼ dish: vegetables and fruits; seasoning: sources of good fats |
| 8) Examples of foods rich in good fats are: | Cream, béchamel, lard | **Avocados, dried fruits, extra virgin olive oil** | Pasta and bread |
| 9) Physical activity in children and adolescents is associated with: | Overweight and obesity | Elevated bad cholesterol | **Better bone, cardiovascular, mental, and cognitive health** |
| 10) Which of these factors related to the environment may negatively affect during mealtime: | Warm, dim lighting | **Crowded environments** | Background music |
| 11) Which of these strategies may be helpful in meal planning? | **Use of pictures and photographs to vary food choices** | Allowing free space for activities before and after the meal | Always suggest the same foods |
| 13) Which of the following factors related to the aesthetics of the meal may be difficult to accept? | Temperatures that are too cold or too high | Use of the same cutlery, the same plate, and the same glass | **Presence of many foods on the same plate** |
| 13) Oral tactile sensory threshold is generally: | **Lower than the rest of the body** | Higher than the rest of the body | Overlapping with the rest of the body |
| 14) Food refusal is a strategy: | **Active** | Passive | Not a strategy |
| 15) Filling your mouth with food may be a symptom of an oral sensory threshold | **High** | Low | Mixed |
| 16) To “get acquainted” with food, one could: | **Prepare together** | Force it to open its mouth | Let it go |
| 17) Eating is: | **A behavior that is learned** | Instinctive | A game |
| 18) The “finger food” stage is essential to: | Explore | Getting to know the food | **All of the above** |
| 19) Food selectivity can be: | By texture alone | By sight, smell and taste | **All of the above** |
| 20) Which therapies are most effective in eating disorder in autism spectrum disorders? | Medical therapies | **Sensory processing, cognitive behavioral, and parent-mediated therapies** | ABA therapy |
| 21) Food refusal may depend on: | Sensory processing difficulties | Cognitive-behavioral difficulties | **All of the above** |

*The answers in bold are the correct ones.*
