# Supplementary material for: Improving Meal Acceptance of Individuals With Autism Spectrum Disorder (AUT-MENU Project): Protocol for a Bicentric Intervention Study
Source: JMIR Res Protoc. 2025 May 21;14:e57507. doi: 10.2196/57507 (PMC12138289; doi:10.2196/57507)
Supplement: Multimedia Appendix 8 [file resprot_v14i1e57507_app8.docx]

| INFORMATION ABOUT YOU | | | | | |
| --- | --- | --- | --- | --- | --- |
| 1) Parent/Caregivers of: | | □ Son  □ Daughter | | | |
| 2) How old is your child: | | ______ | | | |
| 3) Attended Institute | | □ Dosso Verde Pavia  □ Dosso Verde Milano  □ Fondazione Tiglio | | | |
| 4) How many modules of the nutrition course did you attend? | | □ Modul 1  □ Modul 2  □ Modul 3  □ Modul 4  □ Modul 5  □ Modul 6 | | | |
|  | | | | | |
| NUTRITION EDUCATION COURSE EVALUATION | | | | | |
|  | **Not at all** | | **Slightly** | **Quite** | **A lot** |
| COURSE ORGANIZATION: |  | |  |  |  |
| 1. Do you think the course was well organized? | □ | | □ | □ | □ |
| 2. Did you feel involved during the course lectures? | □ | | □ | □ | □ |
| 3. Did you find the online mode to your liking? | □ | | □ | □ | □ |
| 4. Would you prefer the in-person mode?  □ Yes  □ No |  | |  |  |  |
|  |  | |  |  |  |
| COURSE CONTENTS: |  | |  |  |  |
| 1. In your opinion, was the course content helpful in reducing your stress during mealtime? | □ | | □ | □ | □ |
| 2. In your opinion, was the course content helpful in reducing your son/daughter's stress during mealtime? | □ | | □ | □ | □ |
| 3. In your opinion, was the course helpful in improving your knowledge of proper nutrition? | □ | | □ | □ | □ |
| 4. In your opinion, was the course helpful in increasing/improving your knowledge about food selectivity and its management? | □ | | □ | □ | □ |
| 5. In your opinion, was the course helpful in improving/consolidating the eating habits of the household? | □ | | □ | □ | □ |
| 6. In your opinion, was the course helpful in expanding your son/daughter's dietary diversity? | □ | | □ | □ | □ |
|  |  | |  |  |  |
| SUGGESTIONS: |  | |  |  |  |
| 1. Do you think there are any aspects to be improved in this course?  □ Yes  □ No |  | |  |  |  |
| 2. If yes, could you indicate which ones?  ______________________________________________________________________________________________________________________________  ______________________________________________________________________________________________________________________________  ______________________________________________________________________________________________________________________________  ______________________________________________________________________________________________________________________________  ______________________________________________________________________________________________________________________________ | | | | | |
