# Supplementary material for: Improving Meal Acceptance of Individuals With Autism Spectrum Disorder (AUT-MENU Project): Protocol for a Bicentric Intervention Study
Source: JMIR Res Protoc. 2025 May 21;14:e57507. doi: 10.2196/57507 (PMC12138289; doi:10.2196/57507)
Supplement: Supplementary file 8 [file resprot_v14i1e57507_app6.docx]

Finanziato dall'Unione europea NextGenerationEU
Ministero dell'Università e della Ricerca
Italiadomani
UNIVERSITÀ DI PAVIA
onfoods
AUT menu

PICTORIAL DIETARY ASSESSMENT TOOL - AUTMENU PROJECT

DATE: ________________ ☐ T0 ☐ T1 OPERATOR: ________________________

SURNAME: _______________________ NAME: ___________________________

PLACE:

Dosso Verde Milano
Dosso Verde Pavia
Tiglio Fondazione Onlus

QUANTITATIVE SECTION

LUNCH	NULL	¼	½	¾	ALL
FIRST COURSE					
MAIN COURSE					
SIDE DISH					
BREAD GRISSINI CRACKERS					
FRUIT YOGURT ICECREAM					
BEVERAGES					

Finanziato dall'Unione europea NextGenerationEU · Ministero dell'Università e della Ricerca · Italiadomani · UNIVERSITÀ DI PAVIA · onfoods · AUT menu

QUALITATIVE SECTION

LUNCH	GENERAL INFOS	COLOUR	TASTE/SMELL	TEXTURE	TEMPERATURE
**FIRST COURSE**	Name: ____________________________<br>Pasta type: ____________________<br>☐ Short (e.g. penne, fusilli, rice, barley)<br>☐ Long (e.g. spaghetti, fettuccine)<br>Grated cheese?<br>☐ Yes<br>☐ No<br>Spices/herbs:<br>☐ Yes<br>☐ No<br>If yes, please specify:<br>________________	☐ White<br>☐ Beige<br>☐ Brown<br>☐ Green<br>☐ Yellow<br>☐ Orange<br>☐ Red<br>☐ Monochrome<br>☐ Bicolor<br>☐ Multicolor	☐ Faint<br>☐ Intense	☐ Dry<br>☐ Firm/Al dente<br>☐ Soft<br>☐ Sticky/Too dry<br>☐ Untied<br>☐ Broth<br>☐ Dense/creamy<br>☐ Liquid<br>☐ Firm/Al dente<br>☐ Soft	☐ Hot<br>☐ Cold<br>☐ Room temperature
**MAIN COURSE**	Name: _______________________<br>____________________________<br>Spices/herbs:<br>☐ Yes<br>☐ No<br>If yes, please specify:<br>______________<br>Breadcrumbs/grating:<br>☐ Yes<br>☐ No	☐ White<br>☐ Beige<br>☐ Brown<br>☐ Green<br>☐ Yellow<br>☐ Orange<br>☐ Red/Pink<br>☐ Monochrome<br>☐ Bicolor<br>☐ Multicolor	☐ Faint<br>☐ Intense	☐ Crunchy<br>☐ Firm/Hard<br>☐ Soft<br>☐ Too dry/chewy	☐ Hot<br>☐ Cold<br>☐ Room temperature
**SIDE DISH**	Name: _______________________<br>☐ Raw<br>☐ Cooked<br>Spices/herbs:<br>☐ Yes<br>☐ No<br>If yes, please specify:<br>______________<br>Breadcrumbs/grating:<br>☐ Yes<br>☐ No	☐ White<br>☐ Beige<br>☐ Brown<br>☐ Green<br>☐ Yellow<br>☐ Orange<br>☐ Red/Pink<br>☐ Monochrome<br>☐ Bicolor<br>☐ Multicolor	☐ Faint<br>☐ Intense	☐ Crunchy<br>☐ Soft<br>☐ Stringy	☐ Hot<br>☐ Cold<br>☐ Room temperature
**BREAD GRISSINI CRACKERS**	☐ Bread<br>☐ Focaccia<br>☐ Crackers<br>☐ Grissini<br>Whole grains?<br>☐ Yes<br>☐ No	☐ White/Beige<br>☐ Brown	☐ Faint<br>☐ Intense	☐ Crunchy<br>☐ Soft	☐ Hot<br>☐ Cold<br>☐ Room temperature
**FRUIT YOGURT ICECREAM**	☐ Fruit<br>Kind: ___________<br>☐ Whole<br>☐ Cutted<br>☐ Fruit puree<br>☐ Raw<br>☐ Cooked<br>☐ With peel<br>☐ Whitout peel<br>☐ Yogurt<br>☐ Icecream<br>☐ Pudding<br>Flavour:<br>______________<br>*(Yogurt: fuits, plain, flavored; Pudding: cream/chocolate; Icecream: creams/fruits)*	☐ White<br>☐ Beige<br>☐ Brown<br>☐ Green<br>☐ Yellow<br>☐ Orange<br>☐ Red/Pink<br>☐ Purple<br>☐ Monochrome<br>☐ Bicolor<br>☐ Multicolor	☐ Faint<br>☐ Intense	☐ Crunchy<br>☐ Soft<br>☐ Creamy<br>☐ Gelatinous	☐ Hot<br>☐ Cold<br>☐ Room temperature
**BEVERAGES**	☐ Water<br>☐ Fruit juice<br>How many glasses? _________	If juice, please specify the flavour:<br>________________	☐ Faint<br>☐ Intense	☐ Liquid	☐ Cold<br>☐ Room temperature
